# Gut microbiota profiling in obese children from Southeastern China

**DOI:** 10.1186/s12887-024-04668-4

**Published:** 2024-03-18

**Authors:** Jingjing Wang, Peifeng Zhuang, Bin Lin, Haiqing Li, Jinlu Zheng, Wenlin Tang, Wenbin Ye, Xiangjian Chen, Mingping Zheng

**Affiliations:** 1 Department of Pediatrics, Ningde Municipal Hospital of Ningde Normal University, Ningde, China; 2 Clinical Medicine Depeatmant of Fujian Medical University, Fuzhou, China; 3 Department of Joint Surgery and Sports Medicine, Ningde Municipal Hospital of Ningde Normal University, Ningde, China

**Keywords:** Obese children, 16S rRNA sequencing, Microbiota profiling, LEfSe analysis, PICRUSt analysis

## Abstract

Childhood obesity not only has a negative impact on a child's health but is also a significant risk factor for adult obesity and related metabolic disorders, making it a major global public health concern. Recent studies have revealed the crucial role of gut microbiota in the occurrence and development of obesity, in addition to genetic and lifestyle factors. In this study, we recruited 19 normal-weight children and 47 children with varying degrees of obesity. A questionnaire survey was conducted to inquire about the family background, lifestyle habits and dietary composition of the 66 children. Findings indicate that fathers of obese children tend to be obese themselves, while children with highly educated mothers are more likely to maintain a normal weight. Furthermore, overweight children tend to spend more time on electronic devices and less time on physical activities compared to their normal-weight counterparts. Obese children exhibit significant differences in breakfast and dinner dietary composition when compared to children with normal weight. Additionally, the gut microbiota of these 66 children was analyzed using 16S rRNA sequencing. Analysis of gut microbiota composition showed similar compositions among children with varying degrees of obesity, but significant differences were observed in comparison to normal-weight children. Obese children exhibited a reduced proportion of Bacteroidota and an increased proportion of Firmicutes, resulting in an elevated Firmicutes/Bacteroidota ratio. Moreover, Actinobacteriota were found to be increased in the gut microbiota of children with varying degrees of obesity. PICRUSt analysis indicated significant metabolic differences in the microbiota functions between obese and normal-weight children, suggesting the composition of gut microbiota could be a crucial factor contributing to obesity. These findings provide valuable insights for the treatment of childhood obesity.

**Supplementary Information:**

The online version contains supplementary material available at 10.1186/s12887-024-04668-4.

## Introduction

In recent years, the global economy's evolution, combined with shifts in dietary habits among other factors, has led to a startling surge in the prevalence of childhood obesity worldwide, considered a serious global health concern. Between 1975 and 2016, the rate of overweight and obesity among children and adolescents aged 5–19 rose from 4% to over 18% [1]. According to the World Obesity Federation Childhood Obesity Atlas Report in 2019, approximately 254,000,000 children and adolescents globally ranging from 5 to19 years old are projected to have obesity by 2030. In China, the report on Chinese residents’ nutrition and chronic diseases highlights that in 2019, the incidence of overweight and obesity in children under the age of 6 was reported to be at 10.4%. Furthermore, in the age group of 6–17 years, the prevalence was found to be at 19%, marking a significant increase of three percentage points from data gathered in 2015 (Bureau of Disease Prevention and Control, National Health and Wellness Commission of the PRC 2022). Obesity led to the emergence of multiple serious obesity-related comorbidities [2], and holds the potential to adversely impact virtually every system within the human body [3, 4]. Children with obesity are at an increased risk of hyperinsulinemia [5], musculoskeletal problems [6] idiopathic intracranial hypertension [7, 8] and other disease. Thus, obesity has escalated into one of the pressing public health crises of the current era.

Even though the precise cause of obesity remains unclear, yet it is believed to stem from a complex interplay of both internal and external factors of an individual, including genetic factors, Endocrine Disorders, psychological depression and eating habits [9, 10]. The gastrointestinal tract is home to a vast array of microbes, boasting a gene pool that significantly outnumbers that of the host [11]. Certain microorganisms within this ecosystem may possess the ability to foster energy storage, while others could potentially encourage leanness, thus indicating potential links to excessive weight gain [12–14]. Recent research has pointed to significant differences in the composition of the gut microbiota between obese and non-obese children [15, 16]. Several investigations involving obese mice and adults have demonstrated an elevated prevalence of Firmicutes and a concurrent decrease in Bacteroidetes within the obese cohort. This shift, resulting in a significantly higher Firmicutes to Bacteroidetes ratio compared to their normal-weight counterparts, is anticipated to serve as a potential obesity biomarker [17–20]. In Belgium, quantitative real-time PCR was performed to analyze the gut microbiota in children aged 6–16. The findings indicated that in comparison to their normal-weight counterparts, obese children exhibited an elevated Firmicutes/Bacteroidetes ratio in their intestinal tract [21]. Recent study was conducted in China employed 16S rRNA gene sequencing to analyze the gut microbiota composition in both obese and normal-weight children. The findings showed that there was a significant reduction in the relative abundance of Bacteroidetes at the phylum level. However, no significant alterations were observed in the levels of Firmicutes, nor were there substantial changes in the Firmicutes/Bacteroidetes ratio [16]. The findings also revealed that in comparison to their normal-weight counterparts, obese children exhibited a decline in gut microbiota diversity and alterations in bacterial community structure. At the genus level, there was a significant increase in the relative abundances of Faecalibacterium, Phascolarctobacterium, Lachnospira, Megamonas, and Haemophilus within the gut, whereas the relative abundance of Oscillospira and Dialister were significant decreased [16]. Numerous investigations have revealed varying outcomes regarding the shifts in Firmicutes and Bacteroidetes within the gut microbiota of obese children, which could be attributed to a multitude of factors such as ethnicity, dietary habits, and lifestyle of the subjects under study [13, 21, 22]. The association between the Firmicutes/Bacteroidetes ratio and childhood obesity necessitates further exploration. However, it is certain that there are significant changes in the gut microbiota of obese children compared to those with normal weight. However, the patterns within these changes are currently unclear and require further exploration.

In order to investigate the causes of childhood obesity, we recruited 66 children from a same geographic area, including 47 children with different degree of obesity and 19 weight-normal children. We conducted a detailed collection and analysis about the educational level and the weight status of the children's parents, as well as the frequency and duration of the children's physical activities, and the time they spent on electronic devices, and dietary composition. The results of these surveys suggest that there is a significant positive correlation between the adoption of healthy daily lifestyle habits and the prevention of obesity in children. Further, a good dietary composition is crucial for managing children's weight. And Highthroughput sequencing of the 16S rRNA gene was used to characterize the composition and diversity of the complex intestinal microbial community. Not only the composition of the gut microbiota in obese children was investigated, but the gut microbiota of children with varying degrees of obesity was also analyzed. Thus, we gained an in-depth understanding of the composition of gut microbiota in children with varying degrees of obesity. Significant changes in the Firmicutes/Bacteroidetes ratio was observed and determined increased abundance of Actinobacteria in the gut microbiota of obese children, a finding that has been scarcely reported in previous research on obesity. The insights gained from our findings may serve as a valuable reference in the prevention and treatment strategies for childhood obesity.

## Material and method

### Study cohort and sample collection

This case-controlled study was conducted in Ningde City, China. The obese cohort was comprised of children aged 6 to 16, who were selected from patients at the Pediatrics Department of Ningde City Hospital between February 2019 and February 2023. The control group was composed of volunteers with a normal Body Mass Index (BMI), who were classified as being in a healthy condition.

The diagnostic criteria for obesity and overweight are based on the "Expert consensus on diagnosis, assessment, and management of obesity in Chinese children" established by Chinese Society of Pediatric Endocrinology and Metabolism in 2022. While the grading of obesity for this study was determined based on the guidelines set forth by the Chinese Medical Nutrition Therapy Expert Consensus on Overweight/Obesity in Children (2016 edition). Accordingly, childhood obesity was classified using the growth-reference standard for body weight proposed by the World Health Organization (WHO). In this classification, children with the same height and good nutritional status are assigned a reference weight of 100%, with a normal range of ± 10% of the reference weight. Children whose weight exceeded 15% of the reference weight were classified as overweight; those with > 20% as mild obesity; those with > 30% as moderate obesity; and those with > 50% as severe obesity.

The study participants were carefully selected based on the following exclusion criteria: (1) diagnosis of renal, endocrine, genetic metabolic, or central nervous system diseases; (2) administration of antibiotics within two weeks prior to fecal sample collection, as these could potentially disrupt the balance of gastrointestinal microbiota; (3) exposure to stressful situations such as trauma or severe infection within two weeks prior to fecal sample collection; (4) manifestation of gastrointestinal symptoms, including but not limited to abdominal pain, constipation, or diarrhea; and (5) receipt of any form of vaccination within one month prior to the study, which could potentially alter gut microbiota levels due to the immune response triggered by the vaccine.

### DNA extraction

DNA was extracted and purified from 200 mg of frozen fecal samples using the Omega Stool DNA Kit. The quality of the isolated DNA was then assessed through agarose gel electrophoresis, and the concentration was quantified using a NanoDrop spectrophotometer.

### PCR amplification and sequencing

Then the V3-V4 variable regions of bacterial 16S rRNA gene were PCR-amplified using the primers (341F 5’-CCTACGGGNGGCWGCAG-3’, 806R (5’-ACTACNVGGGTWTCTAAT-3’) following the PCR program 95 ℃, for 5 min, 28 cycles at 95 ℃ for 45 s, 55 ℃ for 50 s, and 72 ℃ for 45 s, with a fnal extension of 72 ℃ for 10 min. All primers contained an 8-nucleotide barcode sequence unique to each sample. All reactions were performed in triplicate in 25 μl volumes containing 12.5 μl 2xTaq Plus Master Mix, 3 μl BSA(2 ng/μl), 4 μl of 2.5 mM dNTPs, 1 μl of each primer (5 μM), and 2 μl template DNA (30 ng DNA). Amplicons were extracted from 2% agarose gels and purifed using an AxyPrep DNA Gel Extraction Kit (Axygen Biosciences, Union City, CA, USA) according to the manufacturer’s instructions. The PCR products were vertified by size on 1.5% agarose gel electrophoresis and purified by Agencourt AMPure XP kit. Raw sequences, deposited in the NCBI BioProject database under the BioProject accession number PRJNA996777.

### Data processing

The raw data was divided into different samples according to the barcode sequence through QIIME (v1.8.0) software. Raw reads were trimmed using Trimmomatic(v.0.36), then reads were spliced using FLASH v1.2.11 with a minimum overlap of 10 nucleotides. After splicing, mothur (v1.44.2) software was used to remove sequences with length less than 200 bp, then the chimeric sequences were removed by uchime method according to the Gold Database. The remaining microbial reads were clustered into features using usearch10 with Unoise3 chimera removal [23] and each feature annotated with the SILVA 138 database with a 99% identity threshold [24]. EasyMicrobiome was use to estimate α-diversity and β-diversity indices based on Bray–Curtis dissimilarity and Unifrac distance, and Linear discriminant analysis Effect Size analysis was applied with LEfSe software [25]. LDA (LDA score ≥ 3) was used to estimate the effect size of each taxon differentially represented in cases and controls. Cladograms, derived from the Linear Discriminant Analysis Effect Size (LEfSe) analysis, were utilized to illustrate the most significantly differentially abundant taxa within the microbiota with the normal group represented in green while the obese group depicted in red. Phylogenetic Investigation of Communities by Reconstruction of Unobserved States (PICRUSt) was used to analyze both 16S rRNA gene relative abundances and the predicted metabolic pathway. The sequences of genes in the merged gene catalogue were aligned to the Kyoto Encyclopedia of Genes and Genomes (KEGG) bioinformatics database.

### Statistical analysis

Statistical analyses were performed depending on the normality of the data, assessed using the PerMANOVA, ANOVA and t-test. Statistical significance was determined at an alpha level of *P* < 0.05.

## Results

### Summary of child obesity survey performed in Ningde

**Fig. 1:**
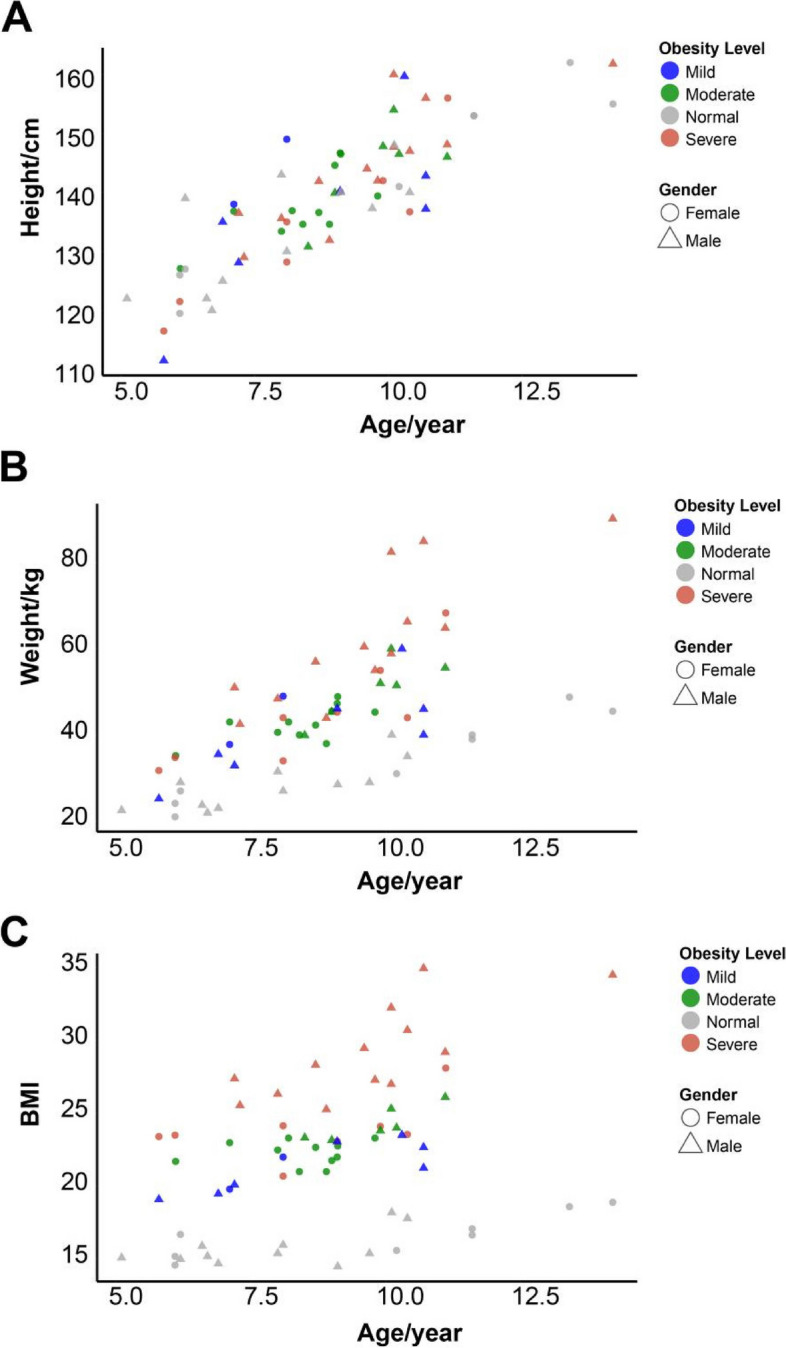
The correlation of physical measurements and age among 66 surveyed children. **A** The correlation of height measurements and age. **B** The correlation of weight measurements and age. **C** The correlation of BMI and age. Blue color represents children in mild obese group, green color represents children in moderate obese group, grey color represents children in normal group, and red color represents children in severe obese group. Triangle shape represents female while round shape represents male

In order to investigate the causes of children obesity, we conducted a survey in 47 individual children with different degree of obesity at the Pediatrics Department of Ningde City Hospital, and 17 volunteers with a normal weight in Ningde city. In this survey, we recorded children’s age, gender, height, weight, and calculated their BMI value (Fig. 1). The 47 individuals with obesity are further categorized into different severity levels: mild, moderate, and severe according to the the guidelines. Among those obese children, there are 9 individuals diagnosed with mild obesity, 17 individuals diagnosed with moderate obesity, and 21 individuals diagnosed with severe obesity (Fig. 1). There were no significant differences in age and height between the obese group and the normal weight group of children (Fig. 1A), Surprisingly, the differences in height and weight between boys and girls are not significant (Fig. 1A and B). Moreover, among children with severe obesity, the proportion of boys is higher than that of girls (Fig. 1C). We conducted a survey on whether the children's parents were overweight and if they had received a university education. We found that the mothers of children with normal weight tended to have a higher level of education, and it is more likely that the fathers of overweight children are themselves overweight. In addition, we also investigated the children's lifestyle habits. Compared with children of normal weight, obese children have lower frequency of exercise per week, spend less time on physical exercise, but more time on electronic devices (Table 1). Furthermore, we conducted a detailed investigation and analysis of the dietary patterns during the three daily meals among children. Comparing obese children to those with normal weight, we observed a lower frequency of meat and egg consumption during breakfast, and a lower frequency of legume and seafood consumption during dinner (Table 2). Diet composition has a significant impact on the composition of gut microbiota [26, 27]. Hence, we hypothesize that good exercise habits and dietary modulation can potentially improve the weight status of obese children.

**Table 1:**
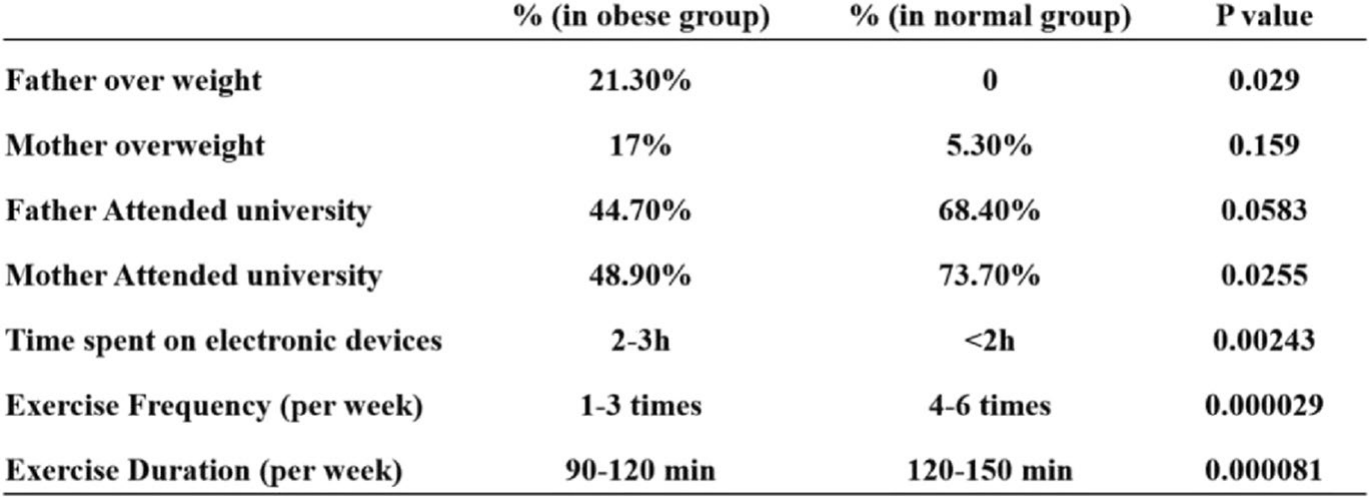
Comparation of the parental weight, educational level, and children’s lifestyle habits between the normal weight group and the obese group

**Table 2:**
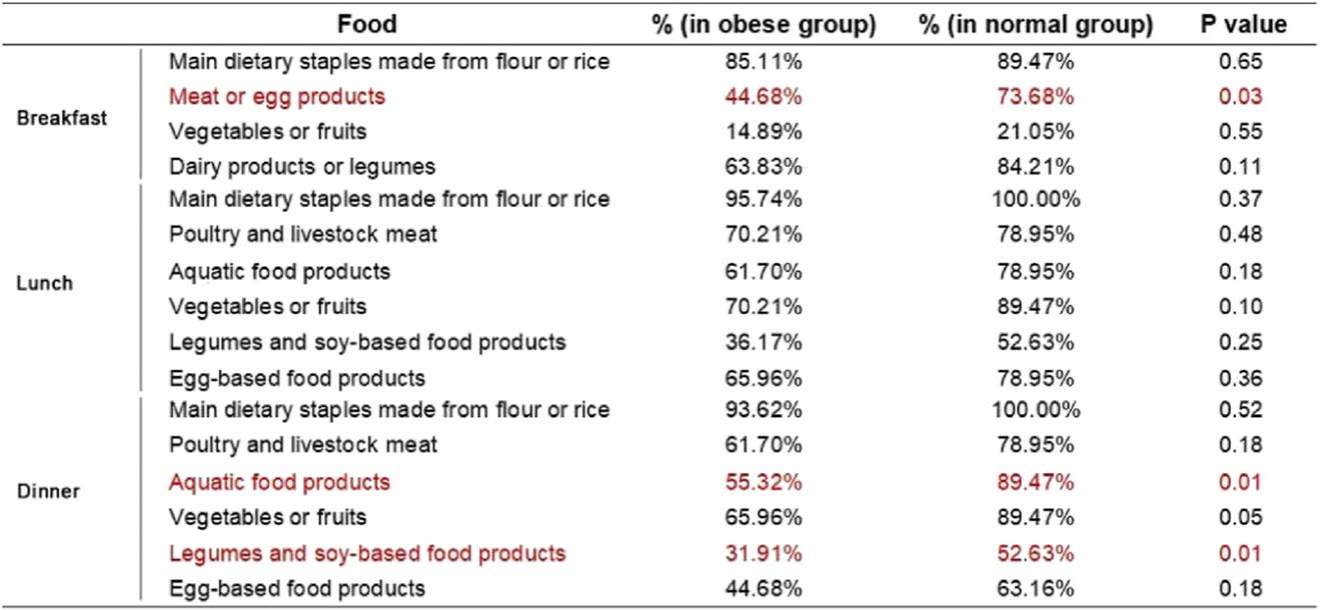
Comparison of the dietary patterns of obese children and normal-weight children in terms of their three daily meals

### Gut microbiota analysis in 66 surveyed children

**Fig. 2:**
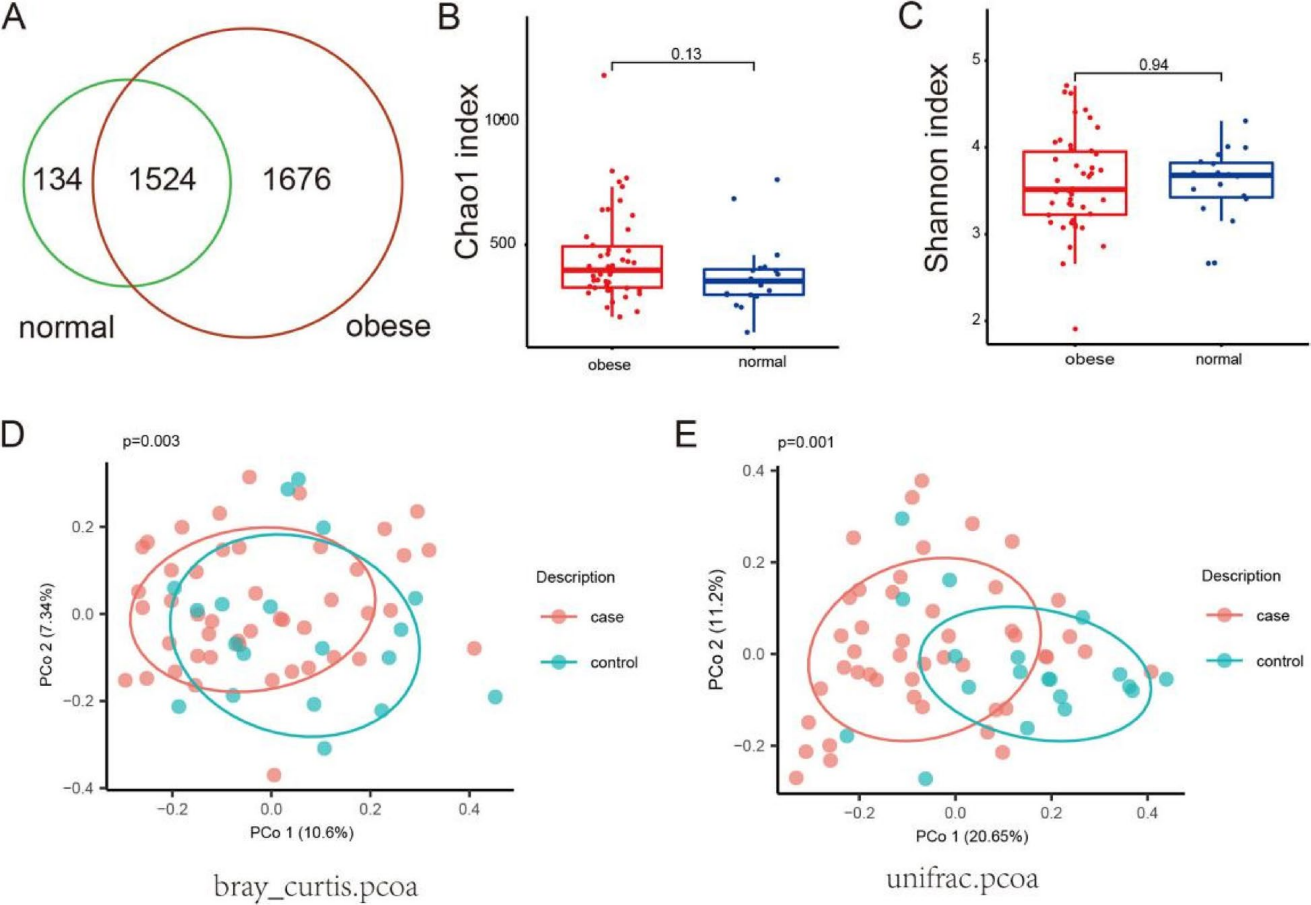
Profiles of gut microbiota in 66 surveyed children. **A** Operational taxonomic units in normal weight group and obese group. **B** Boxplotsfor comparison of Chao1 index between the two study groups. The t-test was used to analyze significant differences. **C** Boxplotsfor comparison of Shannon index between the two study groups. The t-test was used to analyze significant differences. **D** PCoA plots based on Bray–Curtis dissimilarity comparing sample distribution between the two groups. PerMANOVA was used to analyze significant differences. **E** PCoA plots based on Unifrac distance comparing sample distribution between the two groups. PerMANOVA was used to analyze significant differences

To investigate the impact of gut microbiota on children in our survey, we obtained stool samples from those 66 individuals and performed 16S rRNA next-generation sequencing on the stool samples. After applying strict trimming criteria to obtain high quality clean tags, then the tags were clustered into different OTUs based upon similarity. 1658 OTUs were obtained from healthy children and 3200 OUTs were obtained from obese children (Fig. 2A). Among these OTUs, 1676 are found exclusively in obese children, while 134 are unique to healthy children (Fig. 2A). The alpha diversity indices (the Chao1and Shannon index) were used to describe alpha diversity, whereas the difference was not statistically significant (Fig. 2B and C). In addition to the alpha diversity, beta diversity calculated based on the Bray–Curtis dissimilarity and Unifrac distance revealed that the bacterial microbiota of obese group apart from that of control group (Fig. 2D and E). The observations unveiled variations in the gut microbial community structure between the normal weight group and the obese group.

### Taxonomic abundancy differences of gut microbiota between obese children and normal children

**Fig. 3:**
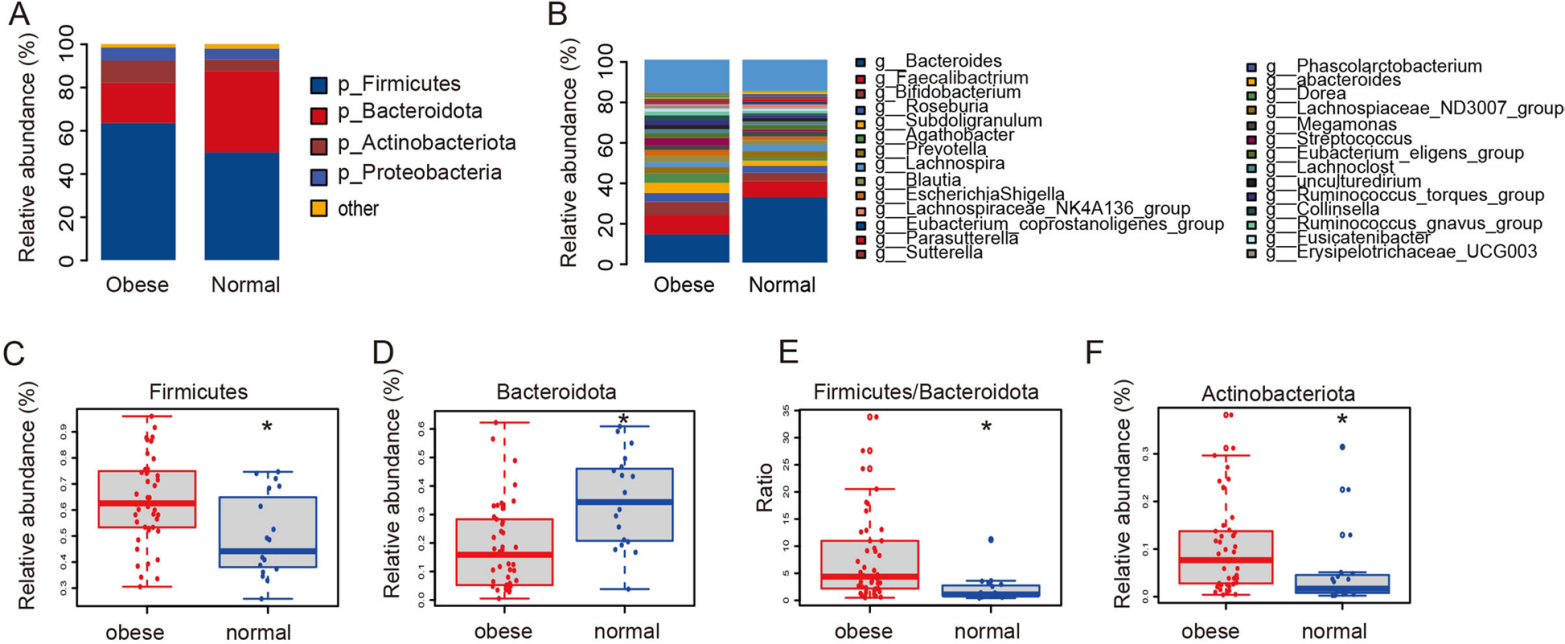
Differences of the community composition of gut microbiota at the phylum and genus level. **A** Profiling of bacterial taxa at the phylum level. **B** Profiling of bacterial taxa at the genus level. Statistical analysis of the differences of Firmicutes (**C**), Bacteroidota (**D**), Firmicutes/Bacteroidota (**E**) and Actinobacteriota (**F**) between the group of obese children and the group of children with normal weight. The t-test was used to analyze significant differences

To further quantify and compare each microbial taxa abundance, we characterized top four phylum: Firmicutes, Bacteroidota, Actinobacteriota, and Proteobacteria. Firmicutes was the most predominant phylum, contributing 49.8% and 63.5% of the gut microbiota in the normal weight group and the obese group, respectively, followed by Bacteroidota, contributing 37.7% and 18.5%, respectively (Fig. 3A and B). Moreover, the abundance of Bacteroidota decreased in the obese group, while the abundance of Firmicutes and Actinobacteriota increased. Sequential genus-level analysis of gut microbiota identified 28 main bacterial taxa in both normal group and obese group (Fig. 3B). More importantly, the comparison between these two groups of children further validated the conclusion of the Firmicutes and Bacteroidota found in phylum-level comparison (Fig. 3B). Statistical analysis showed significant differences in Firmicutes, Bacteroidota, Actinobacteriota, and the Firmicutes to Bacteroidota ratio between obese and normal-weight children. (Fig. 3C-F).

**Fig. 4:**
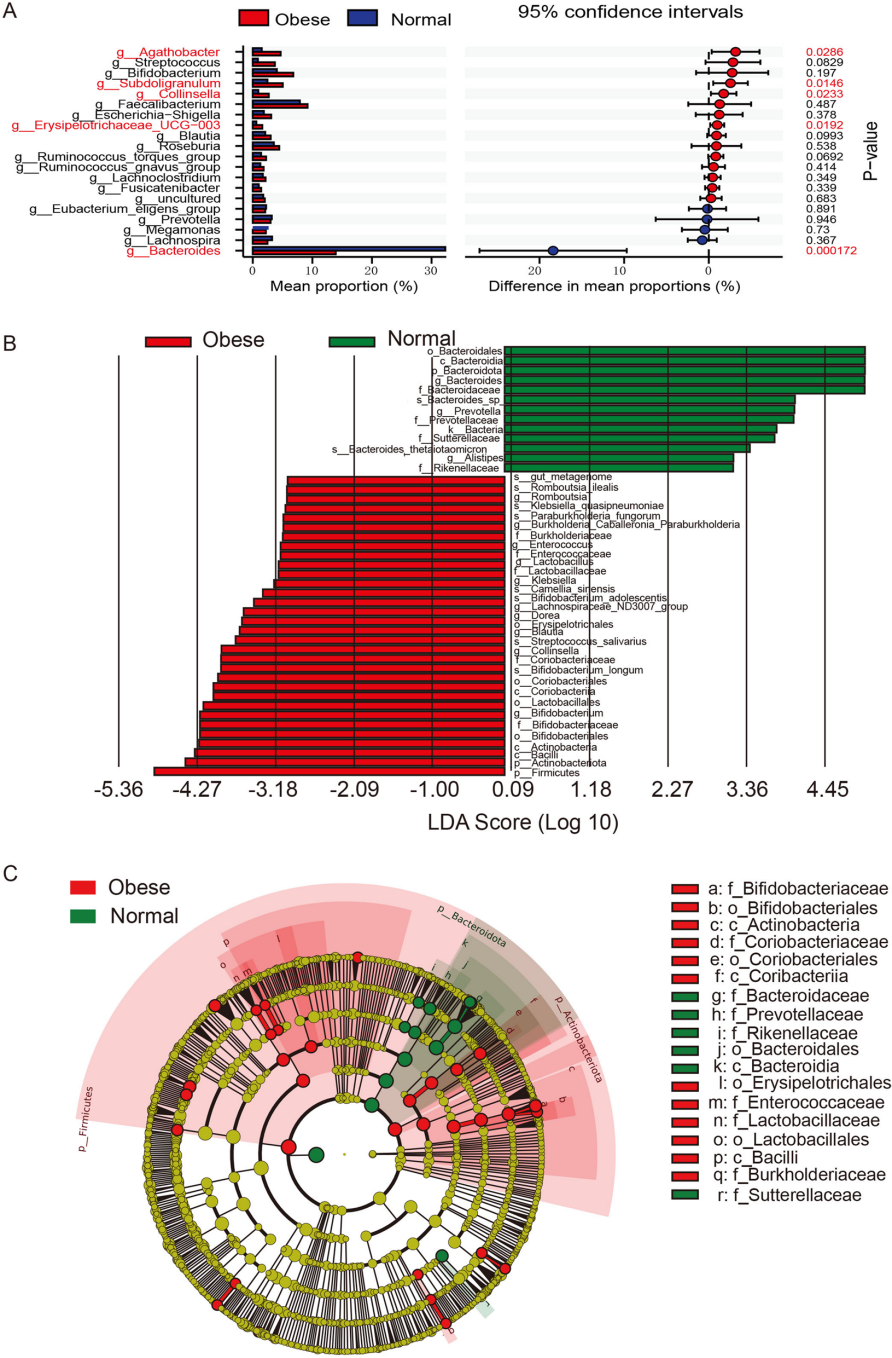
Comparison of gut microbiota structure and abundance using STAMP and LEfSe analysis. **A** Differences between the normal weight group and obese groups were assessed by STAMP anaysis. **B** Histogram of the linear discriminant analysis scores for differentially abundant genera between the two groups. (a logarithmic linear discriminant analysis score > 3 indicated a higher relative abundance in the corresponding group compared to the other group). LDA: Linear discriminant analysis. **C** The taxonomic cladogram obtained from linear discriminant analysis effect size analysis. The diameter of each circle is proportional to the taxon abundance. The dimension of each individual circle is directly proportionate to the abundance of the corresponding taxonomic unit

To meticulously probe the differential composition of microbiota implicated in the progression of childhood obesity, we executed an analysis employing the STAMP to discern the disparities at the genus level between normal-weight and obese cohorts. Among 20 identified genus groups, we found a few microbial groups such as Agatholacter, Collinsella, Erysipelotrichaceae, and Subdoligranulum are significantly enriched in obese children’s gut microbiota (Fig. 4A). On the other hand, Bacteroides are significantly reduced in the obese children group and overly represented in the normal children group (Fig. 4A). Then we further confirmed the differentially abundant taxa by LEfSe, an algorithm for high-dimensional biomarker discovery [25], which identified 45 discriminative features (LDA score ≥ 3) with relative abundance varied significantly between the obese group and control group (Fig. 4B). In addition to variations in Firmicutes, Bacteroidota, and Actinobacteriota, the LEfSe analysis revealed a significant enrichment of Sutterellaceae in the gut microbiota of normal weight children (Fig. 4C). Together, our taxonomic comparison between the gut microbiota of normal children group and that of obese children group reveals certain microbial communities that are related in either normal children group or the obese children group which are potentially important for the future children obesity early diagnosis and management.

### gut microbiota composition among children with mild, moderate, and severe obesity

**Fig. 5:**
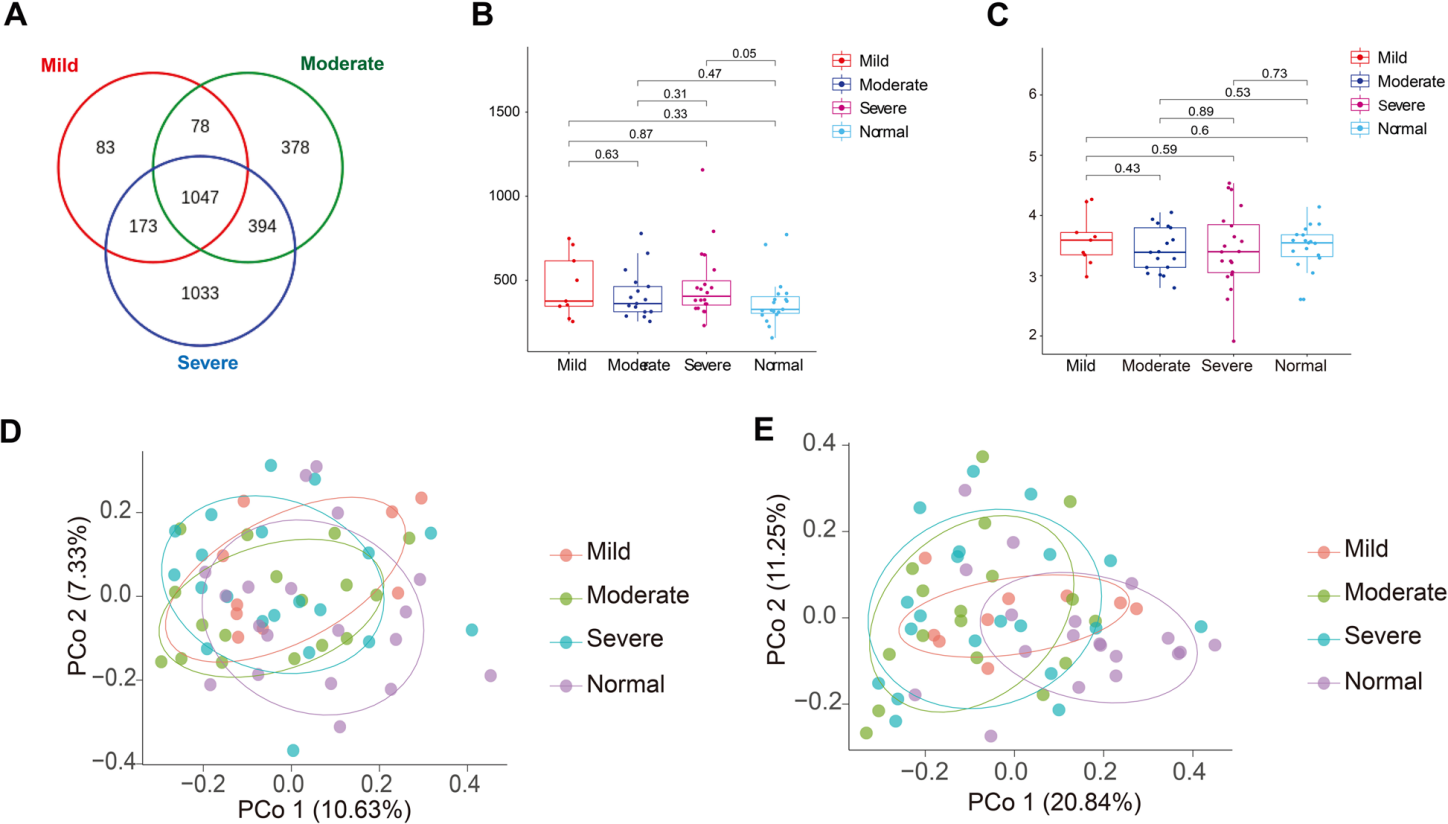
Profiles of gut microbiota in children with mild, moderate, and severe obesity. **A** Operational taxonomic units in group of children with mild, moderate, and severe obesity. **B** Boxplotsfor comparison of Chao1 index among the study groups. ANOVA analysis with FDR correction was used to analyze significant differences. **C** Boxplotsfor comparison of Shannon index among the study groups. ANOVA analysis with FDR correction was used to analyze significant differences. **D** PCoA plots based on Bray–Curtis dissimilarity comparing sample distribution among the study groups. PerMANOVA was used to analyze significant differences. **E** PCoA plots based on Unifrac distance comparing sample distribution among the study groups. PerMANOVA was used to analyze significant differences

We classified obese children into mild, moderate, and severe groups based on guidelines and examined whether gut microbial distribution correlates with various obesity levels. Initially, we compared the OTUs from these three groups. We found that the majority of OTUs from the mild obese group overlapped with those of both moderate and severe obese groups. However, the moderate obese group has 378 unique OTUs and the severe obese group has 1033 unique OTUs compared to the other groups (Fig. 5A). We calculated the alpha diversity indices (Chao1 and Shannon) of the gut microbiota in normal weight children, mildly obese, moderately obese, and severely obese children. We found that the Chao1 index was higher in children with varying degrees of obesity compared to normal weight children. However, only the Chao1 index of severely obese children showed a statistically significant difference from that of normal weight children. No significant differences were observed among other groups (Fig. 5B). The Shannon index was observed to be lower in the groups with moderate and severe obesity compared to the normal weight group, however, the statistical analysis did not show a significant difference (Fig. 5C). Beta diversity analysis found no significant differences amongst the groups with varying degrees of obesity. However, significant differences were identified between the normal weight group and each of the groups with different levels of obesity (Fig. 5D and E). Together, these findings suggest that obesity can lead to an increase in the variety of gut microbiota and induces a significant difference in the composition of the gut microbiota, which is more pronounced in individuals with severe obesity.

**Fig. 6:**
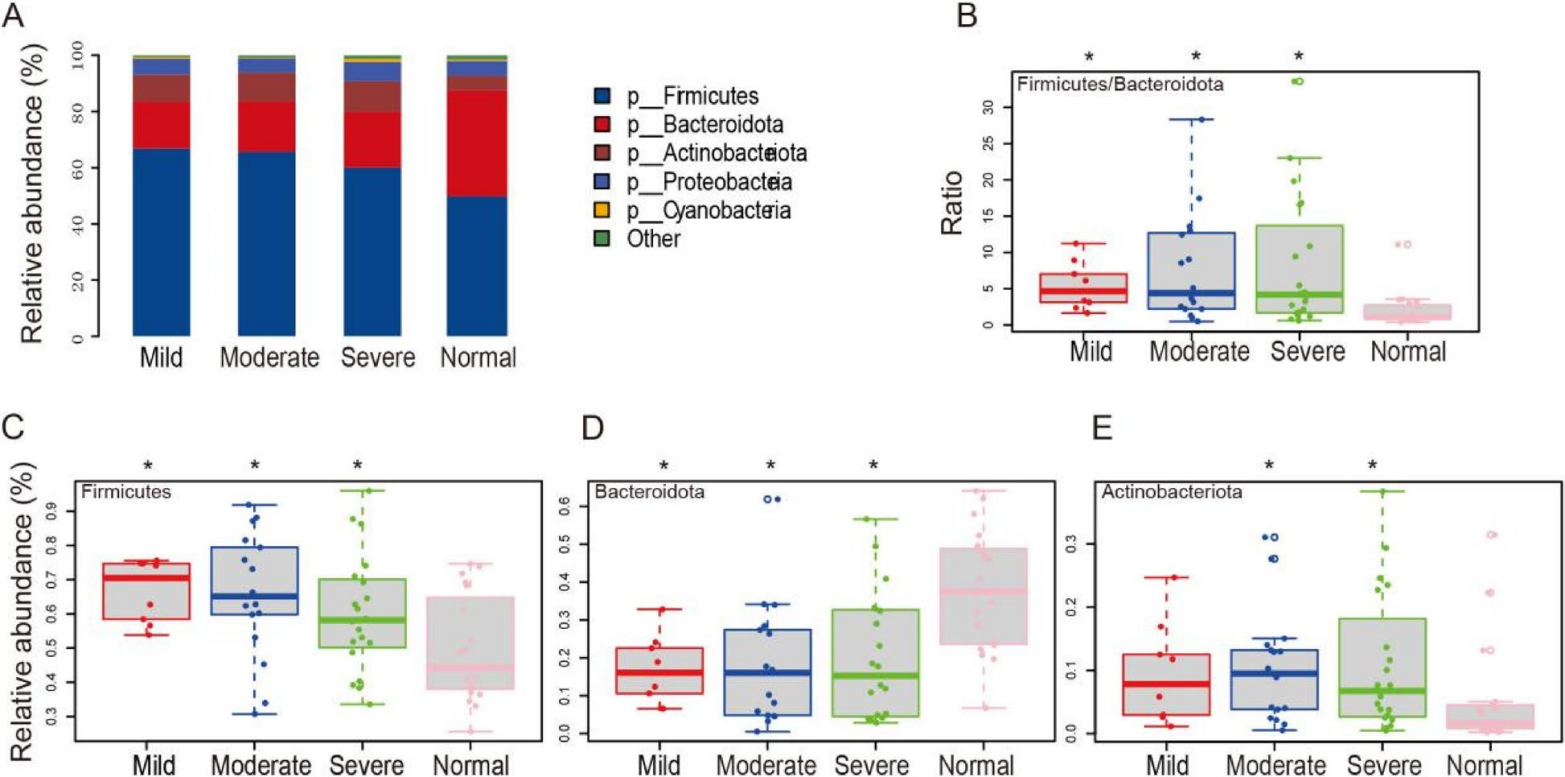
Differences of the community composition of gut microbiota at the phylum level. **A** Profiling of bacterial taxa at the phylum level. Statistical analysis of the differences of Firmicutes/Bacteroidota (**B**), Firmicutes (**C**) and Bacteroidota (**D**) and Actinobacteriota (**E**) among the study groups. The t-test was employed to examine the significant differences between groups of different obesity grades and the control group

To examine the overall composition and diversity of microbial communities, we analysis the bacterial composition at the phylum level (Fig. 6A). Our findings revealed a marked escalation in the Firmicutes to Bacteroidota ratio within the gastrointestinal tract of children spanning various obesity levels, in contrast to their normal-weight counterparts (Fig. 6B). This was accompanied by a pronounced surge in the prevalence of Firmicutes across all levels of obesity, coupled with a notable decrement in Bacteroidota (Fig. 6C and D). Moreover, Actinobacteriota demonstrated a significant proliferation in children categorized under moderate and severe obesity (Fig. 6E). To acquire a more statistical comparison of gut microbiota composition in three obese children groups, we performed STAMP analysis at genus level and we found that Erysipelotrichaceas and Agathobacter were enriched in the moderate obese group rather than in the mild obese group (Figure S1A). When we compared the mild group against serve group, we found that only Erysipelotrichaceas is depleted in the severe obese group than the mild obese group (Figure S1B). Surprisingly, we did not find any microbial genus is significantly enriched or depleted in the comparison of the moderate group against severe group (Figure S1C). These research findings indicate that obesity has a significant impact on altering the composition of the gut microbiota. However, the variation in gut microbiota composition among children is relatively limited across different degrees of obesity.

### Phylogenetic and taxonomic profiles of gut microbiota and PICRUSt analysis of children with varying degrees of obesity

**Fig. 7:**
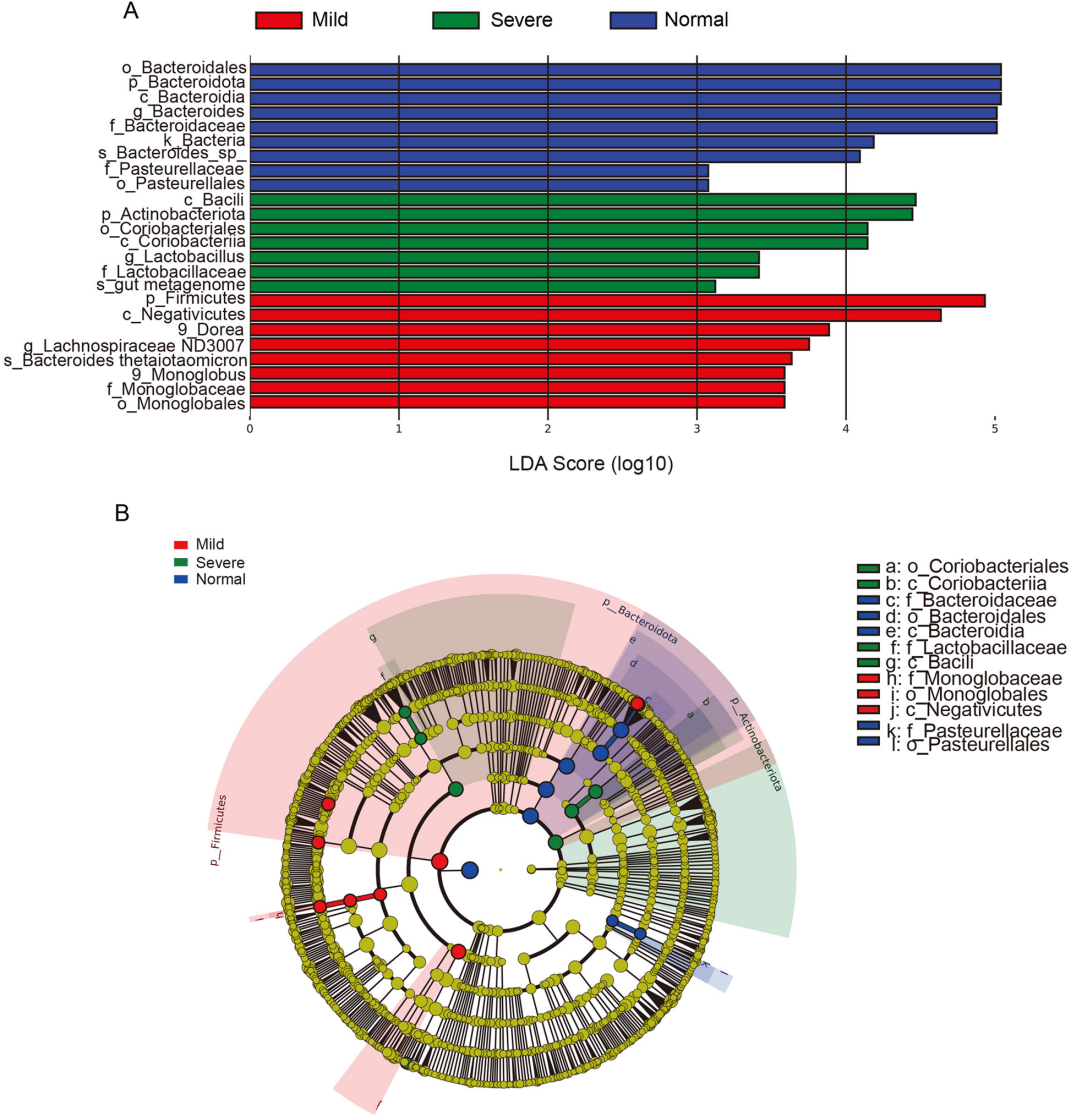
Comparison of gut microbiota structure and abundance using LEfSe analysis among mild, moderate, and severe obesity groups. **A** Histogram of the linear discriminant analysis scores for differentially abundant genera among the study groups. (a logarithmic linear discriminant analysis score > 3 indicated a higher relative abundance in the corresponding group compared to the other group). LDA: Linear discriminant analysis. **C** The taxonomic cladogram obtained from linear discriminant analysis effect size analysis. The diameter of each circle is proportional to the taxon abundance

Given the minimal differences in gut microbiota composition between moderate and severe obese children, we performed LEfSe analysis on data from mildly obese, severely obese, and normal weight children. As shown in Fig. 7A, the relative abundance of taxonomic groups (LDA score ≥ 3) was summed for the normal weight group, mild and severe obese group, and a total of 9 taxa were abundant in the normal weight group, with 8 taxa in the mild obese group and 7 taxa in the severe obese group (Fig. 7A). At the order level, compared to obese children, normal-weight children had higher abundances of Bacteroidales and Pasteurellales in their gut microbiota. In contrast, order Coriobacteriales was more abundant in the gut microbiota of severely obese children, while order Monoglobales was more prevalent in mildly obese children (Fig. 7A). From the cladograms generated from the LEfSe analysis, we found the identified differential taxa mainly originating from three bacterial phyla: Firmicutes, Bacteroidota, and Actinobacteriota. However, we also noted a significant enrichment of order Pasteurellales in children of normal weight, thereby establishing it as a distinguishing biomarker when compared to their obese counterparts (Fig. 7B). In the mild obesity group, the identified biomarkers were class Negativicutes and order Monoglobales, which belong to Firmicutes. In the severe obesity group, the biomarkers identified were class Bacilli, family Lactobacillaceae, and class Coriobacteriia. It is important to note that class Bacilli and family Lactobacillaceae belong to the phylum Firmicutes, whereas class Coriobacteriia belongs to the phylum Actinobacteria.

**Fig. 8:**
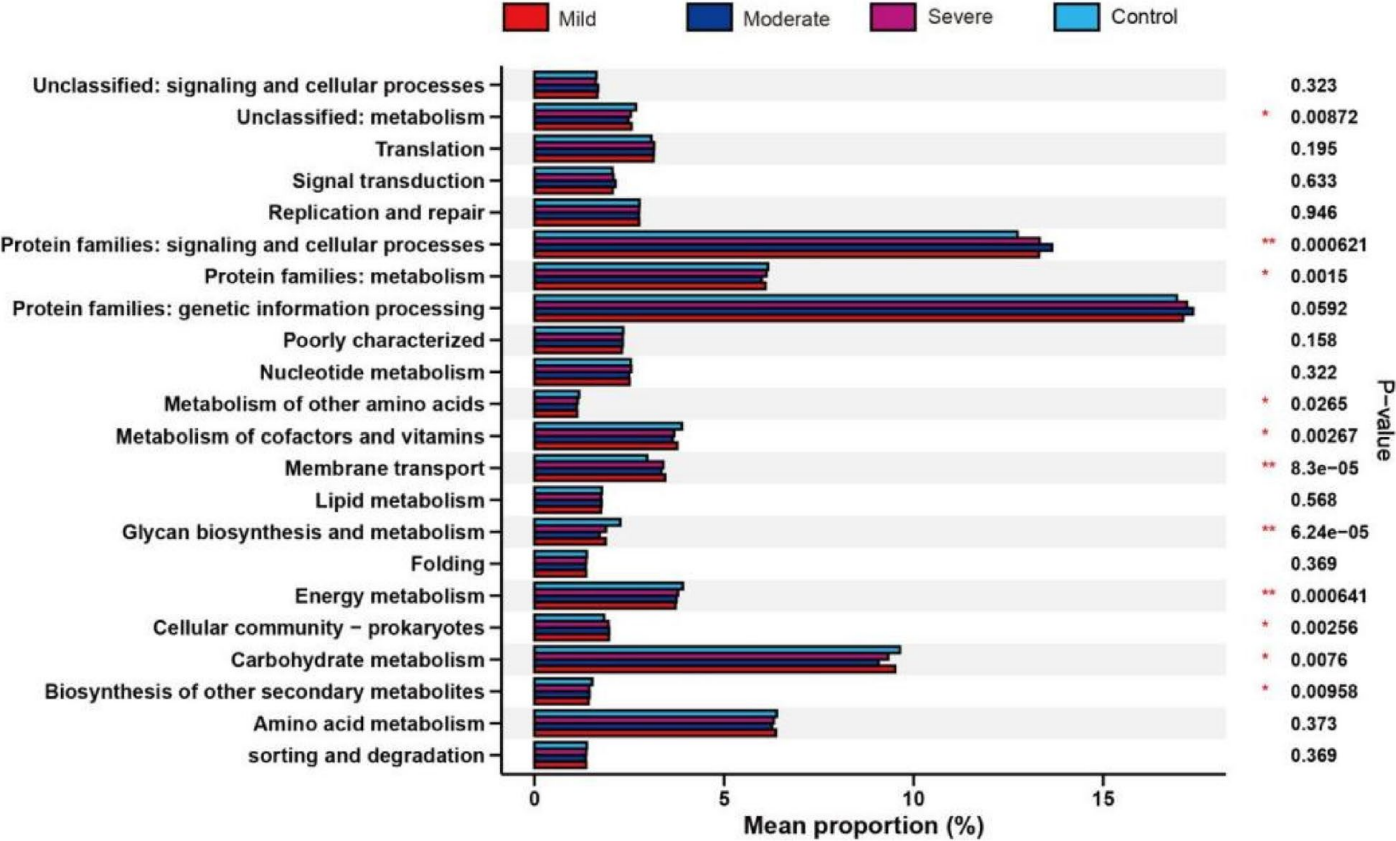
Functional analysis of microbiota in healthy children and children with obesity. PICRUSt analysis showing potential functions of altered gut microbiota. Significantly enriched functions are marked in red

The results of PICRUSt functional prediction (Fig. 8) showed that a total of 22 functional subcategories were identified in normal weight group and obese groups, and revealed that the normal children group have higher level of microbial taxa with the function of metabolism, metabolism of amino acids, glycan biosynthesis and metabolism, energy metabolism, and carbohydrate metabolism (Fig. 8). On the contrary, we also found that the obese children groups have a more enriched bacteria groups whose functions are annotated as signaling and cellular processes and membrane transport (Fig. 8). These findings suggest that alterations in bacterial composition can exert a profound influence on gut microbiota function, particularly in metabolism, which may play a contributory role in the development of childhood obesity.

## Discussion

Childhood obesity has become a significant health concern in China. In order to understand the significance of gut microbiota in child obesity development, we conducted a survey in children in Ningde to monitor obesity trends and assess the causes of children obesity. The findings of the survey revealed a significantly higher proportion of obesity among fathers of obese children compared to those of children with normal weight, suggesting a potential genetic predisposition. Additionally, obese children tend to spend less time engaging in physical activities and more time on electronic devices, implying that a healthy lifestyle is essential for maintaining a normal weight (Table 1). Analysis of the dietary composition during the three daily meals of the children observed certain discrepancies between obese and normal-weight children. Specifically, normal-weight children demonstrated significantly higher frequency intake of meat and egg products during breakfast, and legume and seafood products during dinner (Table 2). Furthermore, mothers, particularly those with higher levels of education, have a substantial influence on their child's lifestyle, with children from these households more inclined to have a healthy weight (Table 1). Moreover, mothers with higher levels of education possess the knowledge to facilitate balanced dietary arrangements for their children. This not only promotes the health of the children but also helps to maintain their normal weight. Further, these dietary practices have an impact not only on the children but also on the weight of their fathers. The types of food consumed have a substantial influence on the composition of the gut microbiota [26]. Hence, there are significant variations in the gut microbiota composition among children with different family backgrounds and dietary habits.

Furthermore, We conducted a comparative study on the composition of gut microbiota between obese and normal-weight children. The alpha diversity, an index that reflects the variety of microbial species within stool samples, is a crucial metric in assessing gut microbiota diversity [28]. Existing research found that the abundance and diversity of gut microbiota in obese children were significantly lower than those in normal weight children [29–31]. However, the alpha diversity indices, including Chao1 and Shannon index of normal and obese group was not statistically significant in this study (Fig. 2B and C). But we observed an upward trend in the Chao1 index and a downward trend in the Shannon index (Fig. 2B and C). The cause of these results may be attributed to the limited size of the study cohort and the considerable heterogeneity among individuals. Beta diversity serves as a metric that encapsulates the variation in the composition of gut microbiota across different samples [28]. In this study, beta diversity analysis indicated the structure of gut microbiota changed significantly with body weight, and these changes may be associated with the occurrence and development of obesity (Fig. 2D and E).

The prevailing consensus on the alterations in the composition of Bacteroidetes and Firmicutes within the gut microbiota remains inconclusive. Some studies have reported a significant decrease in Bacteroidetes accompanied by an increase in Firmicutes, suggesting a potential shift in microbiota composition associated with obesity [32, 33]. However, other research contradicts this, indicating no significant difference in the presence of Firmicutes, despite a marked reduction in Bacteroidetes within the obese group [16, 34]. Interestingly, there are also studies that have observed an increase in the numbers of both Bacteroidetes and Firmicutes in obese children [35]. Our research has found that the proportion of Bacteroidetes is consistently decreased, while the proportion of Firmicutes is consistently increased in obese children (Fig. 3A-E). Additionally, the Firmicutes to Bacteroidetes (F/B) ratio is frequently considered as a potential obesity marker in numerous studies. Particularly, a higher F/B ratio has been observed in obese animals compared to their normal-weight counterparts. However, this specific correlation does not appear to be directly applicable or consistent in human studies [18, 36]. Research conducted on the gut microbiota of obese children residing in the Antwerp region of Belgium and the Kazak region of Xinjiang has revealed a significant elevation in the Firmicutes to Bacteroidetes (F/B) ratio [21]. Our study, conducted in the city of Ningde in Fujian Province, Southeast China, also discovered a significant increase in the F/B ratio in the intestinal tract of obese children, corroborating the findings of the research conducted in Xinjiang (Fig. 3E). Hence, the elevation in the Firmicutes/Bacteroidetes (F/B) ratio appears to be a relatively universal phenomenon within the gut microbiota composition of obese children.

In our research, we further analyzed the gut microbiota composition of children with varying degrees of obesity, discovering a minimal difference in their microbial makeup. Compared to children with normal weight, the proportion of Bacteroidetes decreased, while Firmicutes increased, leading to a significant elevation in the F/B ratio (Fig. 6). The proliferation of Firmicutes within the gut microbiota has been linked to enhanced energy harvest, thereby increasing the overall energy intake [37]. Gut bacteria also possess genes encoding carbohydrate-active enzymes (CAZymes), which are essential for breaking down complex dietary carbohydrates into absorbable components by the intestinal epithelium. This increased efficiency in energy production can contribute to higher energy extraction. Additionally, it has been observed that CAZymes associated with body mass index (BMI) are specifically abundant in the Firmicutes phylum. Moreover, Firmicutes can degrade non-digestible dietary nutrient sources, such as pectin and cellulose. As a result, the higher abundance of Firmicutes is linked to greater energy extraction, potentially leading to increased energy intake [38]. Bacteroidetes serve a crucial function in breaking down plant polysaccharides that are indigestible by the human body, and collaborates with other bacterial species in nutrient metabolism. A sustained high-fat dietary intake may result in a decrease in Bacteroidetes population, thereby disrupting the absorption of polysaccharides and proteins and potentially leading to obesity [39]. Further, Bacteroidetes have been found to possess anti-obesity effects, which can inhibit obesity and promote branched-chain amino acid catabolism in the brown adipose tissue [40], and improve insulin sensitivity and serum glucagon-like peptide-1 [41], which aligns with the phenomenon of an increased ratio of Firmicutes to Bacteroidetes (F/B) in the gut of obese children. The results of the LEfSe analysis also indicate that the abundance of Bacteroidetes and Firmicutes could serve as biological markers to differentiate between children with varying degrees of obesity and those with normal weight (Figs. 4B, C and 7). In addition, we observed a significant increase in the proportion of Actinobacteriota in obese children, an association that has been infrequently explored in previous research (Figs. 3F and 6E). The proportion of Actinobacteria was significantly associated with dietary fat content, and associated with gut barrier impairment, leading to colonic inflammation [42]. So the elevated proportion of Actinobacteria in obesity may be attributed to an imbalanced diet, which can lead to intestinal damage and inflammation.

We employed the LEfSe analysis to identify biomarkers in children with varying degrees of obesity. Children with normal weight exhibited biomarkers belonging to the phyla Bacteroidetes and Proteobacteria. In the mild obesity group, the biomarkers were associated with the phylum Firmicutes, while in the severe obesity group, the biomarkers were associated with both the phyla Firmicutes and Actinobacteria. This finding suggests that in the mild obesity group, there is a pronounced alteration in the proportion of Firmicutes in the gut microbiota, whereas in the severe obesity group, there are significant changes in the proportions of both Firmicutes and Actinobacteria. We speculate that the changes in Actinobacteria may occur downstream of Firmicutes, leading to intestinal damage and inflammation associated with Actinobacteria in the gut. However, the composition of gut microbiota is influenced by various factors including diet, lifestyle habits, race, and geographical location. Our study has limitations in terms of a limited sample size and the analysis was based on a single-time point sampling, therefore, this conclusion warrants further verification and validation through subsequent research. Through the PICRUSt analysis, we have gained insights into how the composition of gut microbiota can influence metabolic functions in the body. Our research provides preliminary evidence and direction for future studies aiming to improve the health of obese children through gut microbiota modulation.

### Supplementary Information

**Additional file 1:** **Supplementary Figure 1.** STAMP analysis of the differernce among the mildly, moderately and severely obese group. (A) Differences between the mildly obese group and moderately obese group were assessed by STAMP anaysis. (B) Differences between the mildly obese group and severely obese group were assessed by STAMP anaysis. (C) Differences between the moderately obese groups and severely obese groups were assessed by STAMP anaysis.

## Data Availability

Raw data are now available at NCBI with BioProject accession PRJNA996777.

## References

[1] Na Z, Guansheng Ma (2018). Childhood obesity in China: trends, risk factors, policies and actions. Global Health J.

[2] Dietz WH, Robinson TN (2005). Clinical practice: overweight children and adolescents. N Engl J Med.

[3] Skinner AC, Perrin EM, Moss LA, Skelton JA (2015). Cardiometabolic risks and severity of obesity in children and young adults. N Engl J Med.

[4] Zuñiga Vinueza AM, Jaramillo AP (2023). The effectiveness of a healthy lifestyle in obese pediatric patients: a systematic review and meta-analysis. Cureus.

[5] Molnár D (2004). The prevalence of the metabolic syndrome and type 2 diabetes mellitus in children and adolescents. Int J Obes Relat Metab Disord.

[6] Chan G, Chen CT (2009). Musculoskeletal effects of obesity. Curr Opin Pediatr.

[7] Brara SM, Koebnick C, Porter AH, Langer-Gould A (2012). Pediatric idiopathic intracranial hypertension and extreme childhood obesity. J Pediatr.

[8] Apperley L, Kumar R, Senniappan S (2022). Idiopathic intracranial hypertension in children with obesity. Acta Paediatr.

[9] Kumar S, Kelly AS (2017). Review of childhood obesity: from epidemiology, etiology, and comorbidities to clinical assessment and treatment. Mayo Clin Proc.

[10] Bertrand-Protat S, Chen J, Jonquoy A, Frayon S, Thu Win Tin S, Ravuvu A, Caillaud C, Galy O (2023). Prevalence, causes and contexts of childhood overweight and obesity in the Pacific region: a scoping review. Open Res Eur.

[11] Gill SR, Pop M, Deboy RT, Eckburg PB, Turnbaugh PJ, Samuel BS, Gordon JI, Relman DA, Fraser-Liggett CM, Nelson KE (2006). Metagenomic analysis of the human distal gut microbiome. Science.

[12] Ignacio A, Fernandes MR, Rodrigues VA, Groppo FC, Cardoso AL, Avila-Campos MJ, Nakano V (2016). Correlation between body mass index and faecal microbiota from children. Clin Microbiol Infect.

[13] Bergström A, Skov TH, Bahl MI, Roager HM, Christensen LB, Ejlerskov KT, Mølgaard C, Michaelsen KF, Licht TR (2014). Establishment of intestinal microbiota during early life: a longitudinal, explorative study of a large cohort of Danish infants. Appl Environ Microbiol.

[14] Scheepers LE, Boonen A, Pijnenburg W, Bierau J, Staessen JA, Stehouwer CD, Thijs C, Arts IC (2017). The intestinal microbiota composition and weight development in children: the KOALA birth cohort study. J Hypertens.

[15] Leong KSW, Jayasinghe TN, Wilson BC, Derraik JGB, Albert BB, Chiavaroli V, Svirskis DM, Beck KL, Conlon CA, Jiang Y, Schierding W, Vatanen T, Holland DJ, O'Sullivan JM, Cutfield WS (2020). Efects of fecal microbiome transfer in adolescents with obesity :the gut bugs randomized controlled trial. JAMA Netw Open.

[16] Chen X, Sun H, Jiang F, Shen Y, Li X, Hu X, Shen X, Wei P (2020). Alteration of the gut microbiota associated with childhood obesity by 16S rRNA gene sequencing. PeerJ.

[17] Patrone V, Vajana E, Minuti A, Callegari ML, Federico A, Loguercio C, Dallio M, Tolone S, Docimo L, Morelli L (2016). Postoperative changes in fecal bacterial communities and fermentation products in obese patients undergoing bilio-intestinal bypass. Front Microbiol.

[18] Ley RE, Bäckhed F, Turnbaugh P, Lozupone CA, Knight RD, Gordon JI (2005). Obesity alters gut microbial ecology. Proc Natl Acad Sci U S A.

[19] Kapoor P, Tiwari A, Sharma S, Tiwari V, Sheoran B, Ali U, Garg M (2023). Effect of anthocyanins on gut health markers, firmicutes-bacteroidetes ratio and short-chain fatty acids: a systematic review via meta-analysis. Sci Rep.

[20] Gong J, Shen Y, Zhang H, Cao M, Guo M, He J, Zhang B, Xiao C (2022). Gut microbiota characteristics of people with obesity by meta-analysis of existing datasets. Nutrients.

[21] Bervoets L, Van Hoorenbeeck K, Kortleven I, Van Noten C, Hens N, Vael C, Goossens H, Desager KN, Vankerckhoven V (2013). Diferences in gut microbiota composition between obese and lean children: a cross-sectional study. Gut Pathog.

[22] Abdallah Ismail N, Ragab SH, AbdElbaky A, Shoeib AR, Alhosary Y, Fekry D (2011). Frequency of Firmicutes and Bacteroidetes in gut microbiota in obese and normal weight Egyptian children and adults. Arch Med Sci.

[23] R.C. Edgar, UNOISE2: improved error-correction for Illumina 16S and ITS amplicon sequencing, bioRxiv, 2016, October 15.

[24] Quast C, Pruesse E, Yilmaz P, Gerken J, Schweer T, Yarza P, Peplies J, Glöckner FO. Nucleic Acids Res. 2013 Jan;41(Database issue):D590-6. 10.1093/nar/gks1219 PMC3531112 23193283

[25] Segata N, Izard J, Waldron L, Gevers D, Miropolsky L, Garrett WS, Huttenhower C (2011). Metagenomic biomarker discovery and explanation. Genome Biol.

[26] Nova E, Gómez-Martinez S, González-Soltero R (2022). The influence of dietary factors on the gut microbiota. Microorganisms.

[27] Zhao Y, Chen L, Yao S, Chen L, Huang J, Chen S, Yu Z (2023). Genome-centric investigation of the potential succession pattern in gut microbiota and altered functions under high-protein diet. Curr Res Food Sci.

[28] Liu R, Hong J, Xu X, Feng Q, Zhang D, Gu Y, Shi J, Zhao S, Liu W, Wang X, Xia H, Liu Z, Cui B, Liang P, Xi L, Jin J, Ying X, Wang X, Zhao X, Li W, Jia H, Lan Z, Li F, Wang R, Sun Y, Yang M, Shen Y, Jie Z, Li J, Chen X, Zhong H, Xie H, Zhang Y, Gu W, Deng X, Shen B, Xu X, Yang H, Xu G, Bi Y, Lai S, Wang J, Qi L, Madsen L, Wang J, Ning G, Kristiansen K, Wang W (2017). Gut microbiome and serum metabolome alterations in obesity and after weight-loss intervention. Nat Med.

[29] Le Chatelier E, Nielsen T, Qin JJ, Prifti E, Hildebrand F, Falony G, Almeida M, Arumugam M, Batto JM, Kennedy S, Leonard P, Li JH, Burgdorf K, Grarup N, Jourgensen T, Brandslund I, Nielsen HB, Juncker AS, Bertalan M, Levenez F, Pons N, Rasmussen S, Sunagawa S, Tap J, Tims S, Zoetendal E, Brunak S, Clement K, Dore J, Kleerebezem M, Kristiansen K, Renault P, Sicheritz-Ponten T, De Vos WM, Zunker JD, Raes J, Hansen T, Bork P, Wang J, Ehrlich SD, Pedersen O (2013). Richness of human gut microbiome correlates with metabolic markers. Nature.

[30] Gao RY, Zhu CL, Li H, Yin MM, Pan C, Huang LS, Kong C, Wang XC, Zhang Y, Qu S (2017). Dysbiosis signatures of gut microbiota along the sequence from healthy, young patients to those with overweight and obesity. Obesity.

[31] Scheithauer TP, Dallinga-Thie GM, De Vos WM, Nieuwdorp M, Van Raalte DH (2016). Causality of small and large intestinal microbiota in weight regulation and insulin resistance. Molecular Metabolism.

[32] Rahat-Rozenbloom S, Fernandes J, Gloor GB, Wolever TMS (2014). Evidence for greater production of colonic short-chain fatty acids in overweight than lean humans. Int J Obes.

[33] Patrone V, Vajana E, Minuti A, Callegari ML, Federico A, Loguercio C, Dallio M, Tolone S, Docimo L, Morelli L (2016). Postoperative changes in fecal bacterial communities and fermentation products in obese patients undergoing biliointestinal bypass. Front Microbiol.

[34] Krajmalnik-Brown R, Ilhan ZE, Kang DW, DiBaise JK (2012). Effects of gut microbes onnutrient absorption and energy regulation. Nutr Clin Pract.

[35] Ismail NA, Ragab SH, Elbaky AABD, Shoeib ARS, Alhosary Y, Fekry D (2011). Frequency of Firmicutes and Bacteroidetes in gut microbiota in obese and normal weight Egyptian children and adults. Archives Med Sci.

[36] Finucane MM, Sharpton TJ, Laurent TJ, Pollard KS. 2014. A taxonomic signature of obesity in the microbiome? Getting to the guts of the matter. PLOS ONE. 2014. 10.1371/journal.pone.0084689 PMC3885756 24416266

[37] Beh BK, Mohamad NE, Yeap SK, Lim KL, Ho WY, Yusof HM, Sharifuddin SA, Jamaluddin A, Long K, Alitheen NB (2016). Polyphenolic profiles and the in vivo antioxidant effect of nipa vinegar on paracetamol induced liver damage. Royal Soc Chemist Adv.

[38] Bhattacharya T, Ghosh TS, Mande SS (2015). Global profiling of carbohydrate active enzymes in human gut microbiome. PLoS ONE.

[39] Murphy EF, Cotter PD, Healy S, Marques TM, O’Sullivan O, Fouhy F, Clarke SF, O’Toole PW, Quigley EM, Stanton C, Ross PR, O’Doherty RM, Shanahan F (2010). Composition and energy harvesting capacity of the gut microbiota: relationship to diet, obesity and time in mouse models. Gut.

[40] Yoshida N, Yamashita T, Osone T, Hosooka T, Shinohara M, Kitahama S, Sasaki K, Sasaki D, Yoneshiro T, Suzuki T, Emoto T, Saito Y, Ozawa G, Hirota Y, Kitaura Y, Shimomura Y, Okamatsu-Ogura Y, Saito M, Kondo A, Kajimura S, Inagaki T, Ogawa W, Yamada T, Hirata KI (2021). Bacteroides spp. promotes branched-chain amino acid catabolism in brown fat and inhibits obesity. iScience.

[41] Yang JY, Lee YS, Kim Y, Lee SH, Ryu S, Fukuda S, Hase K, Yang CS, Lim HS, Kim MS, Kim HM, Ahn SH, Kwon BE, Ko HJ, Kweon MN (2017). Gut commensal Bacteroides acidifaciens prevents obesity and improves insulin sensitivity in mice. Mucosal Immunol.

[42] Kim SJ, Kim SE, Kim AR, Kang S, Park MY, Sung MK (2019). Dietary fat intake and age modulate the composition of the gut microbiota and colonic inflammation in C57BL/6J mice. BMC Microbiol.

