# Supplementary figures and images for: Gut microbiota profiling in obese children from Southeastern China
Source: BMC Pediatr. 2024 Mar 18;24:193. doi: 10.1186/s12887-024-04668-4 (PMC10946167; doi:10.1186/s12887-024-04668-4)

Figure S1


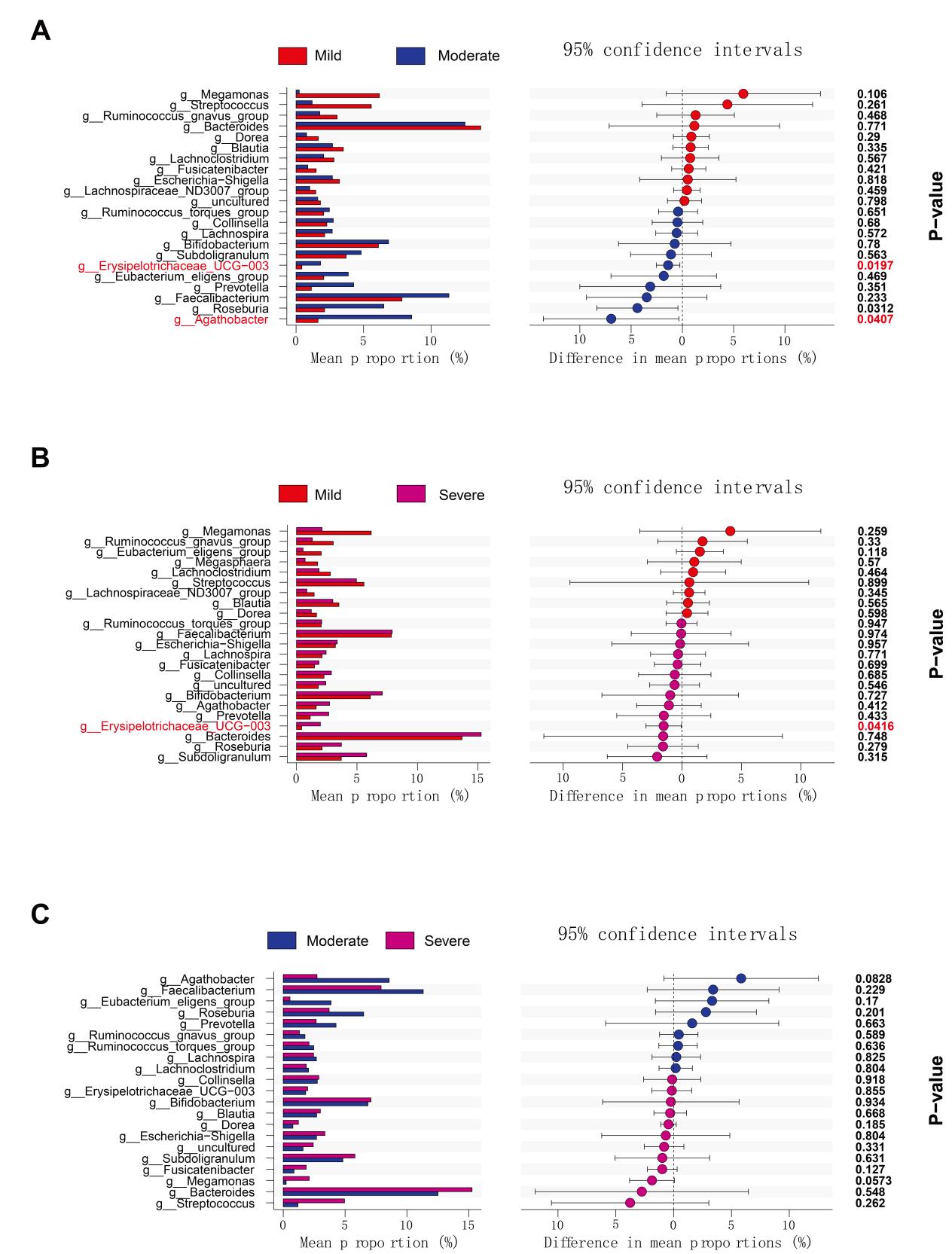

Supplement: Supplementary file 1 — Additional file 1: Supplementary Figure 1. STAMP analysis of the differernce among the mildly, moderately and severely obese group. (A) Differences between the mildly obese group and moderately obese group were assessed by STAMP anaysis. (B) Differences between the mildly obese group and severely obese group were assessed by STAMP anaysis. (C) Differences between the moderately obese groups and severely obese groups were assessed by STAMP anaysis. [file 12887_2024_4668_MOESM1_ESM.docx]
